# Improved Recombinant Adeno-Associated Viral Vector Production via Molecular Evolution of the Viral Rep Protein

**DOI:** 10.3390/ijms26031319

**Published:** 2025-02-04

**Authors:** Thomas Steininger, Veronika Öttl, Linda E. Franken, Cornelius Frank, Philip Ohland, Miriam Lopez Ferreiro, Stefan Klostermann, Johannes Fritsch, Evelyn Hirschauer, Anna Sandmeir, Luisa D. Hilgenfeld, Florian Semmelmann, Marie-Sofie Dürr, Fabian Konkel, Gregor Pechmann, Sabine Linder, Markus Haindl, Mustafa N. Yazicioglu, Philippe Ringler, Matthias E. Lauer, Denis Phichith, Stefan Seeber, Julia Fakhiri

**Affiliations:** 1 Roche Pharma Research and Early Development, Therapeutic Modalities, Roche Innovation Center Munich, Roche Diagnostics GmbH, Nonnenwald 2, 82377 Penzberg, Germany; thomas.steininger.ts1@roche.com (T.S.); veronika.oettl@roche.com (V.Ö.); cornelius.frank@roche.com (C.F.); johannes.fritsch@roche.com (J.F.); evelyn.hirschauer@roche.com (E.H.); fabian.konkel@roche.com (F.K.); sabine.linder@roche.com (S.L.); stefan.seeber@roche.com (S.S.); 2 Faculty of Bioengineering, University of Applied Sciences Weihenstephan-Triesdorf, Am Hofgarten 4, 85354 Freising, Germany; 3 Faculty 06, Munich University of Applied Sciences, Lothstraße 34, 80335 Munich, Germany; 4 Faculty of Medicine, University of Regensburg, Universitätsstraße 31, 93053 Regensburg, Germany; 5 Roche Pharma Research and Early Development, Therapeutic Modalities, Roche Innovation Center Basel, Hoffmann-La Roche Ltd., Grenzacherstrasse 124, 4070 Basel, Switzerland; linda_elise.franken@roche.com (L.E.F.); philip.ohland@roche.com (P.O.); miriam.lopez_ferreiro@roche.com (M.L.F.); philippe.ringler@roche.com (P.R.); matthias.lauer@roche.com (M.E.L.); denis.phichith@roche.com (D.P.); 6 Gene Therapy Technical Research & Development, Roche Diagnostics GmbH, Nonnenwald 2, 82377 Penzberg, Germany; marie-sofie.duerr@roche.com (M.-S.D.); gregor.pechmann@roche.com (G.P.); markus.haindl@roche.com (M.H.); 7 Roche Pharma Research and Early Development, Data and Analytics, Roche Innovation Center Munich, Roche Diagnostics GmbH, Nonnenwald 2, 82377 Penzberg, Germany; stefan.klostermann@roche.com; 8 Technical Development Analytics, Roche Diagnostics GmbH, Nonnenwald 2, 82377 Penzberg, Germany; anna.sandmeir@roche.com (A.S.); luisa_desiree.hilgenfeld@roche.com (L.D.H.); florian.semmelmann@roche.com (F.S.); 9 Spark Therapeutics, Roche Holding AG, 3737 Market Street, Philadelphia, PA 19104, USA; mustafa.yazicioglu@sparktx.com; 10 Biozentrum, University of Basel, Spitalstrasse 41, 4056 Basel, Switzerland

**Keywords:** AAV, rAAV production, directed evolution, replication proteins, protein engineering

## Abstract

In the dynamic field of gene therapy, recombinant adeno-associated viruses (rAAVs) have become leading viral vectors due to their safety, long-term expression, and wide-ranging cell and tissue tropism. With numerous FDA approvals and commercial products underscoring their potential, there is a critical need for efficient production processes to achieve high vector titers and quality. A major challenge in rAAV production is the efficient packaging of the genome into the viral capsid, with empty or partially filled capsids often representing over 90% of the produced material. To tackle this issue, we engineered the replication and packaging proteins of an AAV (Rep) to boost their functionality and improve vector titers. We subjected a complex Rep library derived from the AAV serotypes 1–13 to directed evolution in an AAV producer cell line. After each round of selection, single clones were analyzed, showing enrichment of specific hybrid Rep domains. Comparative analysis of these selected clones revealed considerable differences in their ability to package AAV2-based viral genomes, with hybrid Rep proteins achieving up to a 2.5-fold increase in packaging efficiency compared to their parental counterparts. These results suggest that optimizing *rep* gene variants through directed evolution is an effective strategy to enhance rAAV production efficiency.

## 1. Introduction

Adeno-associated viral (AAV) vectors hold great promise for delivering therapeutic genes. However, the high production costs of AAV gene therapy products represent a significant challenge within the field [1,2]. This is mainly attributed to the complexity of the manufacturing process, which involves intricate production steps to ensure the generation of high-quality viral vectors and their subsequent purification. Empty AAV capsids, which lack the therapeutic gene and can impact the overall efficacy and safety of AAV vectors, are one of several potential contaminants of viral vector preparations. Purification strategies have evolved to separate full from empty capsids, but recent research revealed that empty capsids are not truly empty and may contain small genomic fragments [3,4], thus complicating their separation from the full-length genome-containing viral capsids. This emphasizes the need for a better understanding of genome replication and packaging mechanisms to reduce these unwanted byproducts.

To date, four nascent and overlapping open reading frames (ORFs) have been identified in wild-type AAVs: Rep [5], Cap [5], AAP [6], and MAAP [7]. The expression of the underlying proteins is strictly regulated by using adeno-inducible promoters, leaky ribosomal scanning, and alternative splicing. In the AAV life cycle, the non-structural proteins (Rep) have emerged as multifaceted entities crucial for the viral life cycle. Four overlapping Rep proteins (Rep78, Rep68, Rep52, and Rep40) are expressed from two promoters at map units 5 and 19 [8]. The large Rep proteins, Rep78 and Rep68, govern processes such as replication [9], transcription regulation [10], and site-specific integration [11]. They orchestrate AAV replication by utilizing endonuclease and helicase activities, ensuring the effective amplification of the viral genome within the host cell [12]. The smaller Rep proteins, Rep52 and Rep40, are believed to contribute to propelling the viral genome into pre-formed capsids [13,14].

By regulating AAV transcription, Rep proteins impact the delicate balance between productive replication and maintenance of the latent state, both in the presence and absence of a helper virus infection [10]. Their interactions with viral DNA elements, including *cis* regulatory elements upstream of viral promoters [15] and inverted terminal repeats (ITRs) [16], dictate the fate of the AAV genome. These interactions are driven by the three main protein domains of the Rep protein: (i) the DNA- or origin-binding domain (OBD), which drives Rep binding to dsDNA at the Rep-binding elements (RBEs) located within the ITRs and the p5 promoter [17]; (ii) the helicase/ATPase domain, which is required for DNA replication and packaging of viral ssDNA into the AAV capsids [12,13]; and (iii) the zinc finger and protein kinase A (PKA) inhibitor-like domain. The zinc finger motif is required for cell cycle arrest in the S-phase [18], while the PKA-inhibitor domain seems to interfere with the PKA-sensitive adenovirus replication, preserving AAV2 replication fitness during a co-infection [19]. Understanding these molecular interactions is crucial for manipulating recombinant AAV vectors (rAAVs) for therapeutic purposes. Here, researchers actively explored strategies to modify Rep proteins, modulating their expression and relative levels to enhance vector production. Notably, the fine-tuning of enzymatic activities was achieved by site-directed mutagenesis of protein domains [20], modifying interactions with host factors [21], and optimizing expression profiles [22,23,24].

Importantly, these endeavors go beyond the AAV2 Rep protein (Rep2) and encompass other Rep variants derived from natural sources. The exploration was spurred by pseudotyping, i.e., the ability to cross-package AAV2-based genomes into capsids other than AAV2 [25,26,27]. While Rep2 excels in its AAV2-based system, cross-complementation with Rep proteins from other AAV serotypes [26,28,29,30] has been demonstrated. Some AAV vector preparation titers could even be increased using the respective ITRs belonging to the utilized Rep protein [20,26,31].

In this study, we aimed to improve the rAAV production process by directed evolution of the Rep protein. To this end, we used a high-throughput approach based on DNA family shuffling (DFS) to build a complex library of hybrid Rep variants. This library underwent screening in a producer cell line over multiple rounds to enrich for new Rep variants with improved properties. Our results showcase the diverse abilities of the 13 natural Rep proteins to replicate and package AAV2 ITR-based viral genomes. The molecular evolution of Rep yielded hybrid variants with distinct properties and a delicate enrichment of combinatorial motifs, either increasing rAAV capsid titer or enhancing packaging of the viral genome. We believe that, building on the promise of our library, the outcome of selection can now be further refined by using different selection pressures and leveraging recent advances in machine learning algorithms [7,32], fostering further enhancements that elevate rAAV vector production for more effective gene therapy applications.

## 2. Results

### 2.1. Cloning and Functional Assessment of 13 AAV Rep Proteins

**Figure 1:**
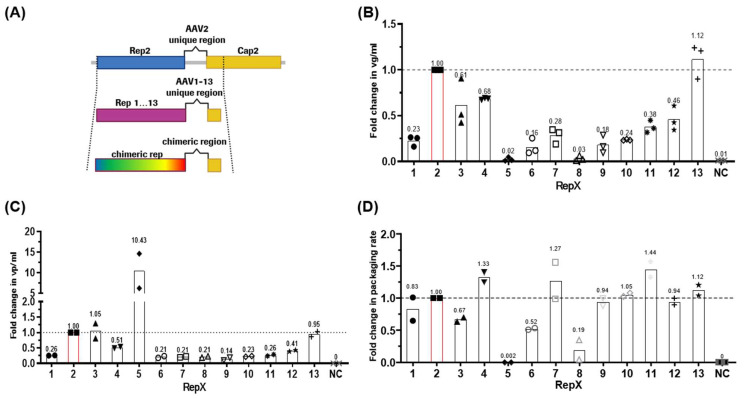
Test of different AAV Rep proteins in rAAV2 production. (**A**) Schematic representation of the cloning strategy for natural *rep* variants 1 to 13 or chimeric *rep* ORFs. The cloning procedure included a unique stretch between *rep* and *cap* in each AAV genome. (**B**) Fold change in viral genomes per ml cell culture lysate (vg/mL). Adherent HEK293T cells were transfected with a rAAV-GFP reporter, an adenohelper plasmid and one plasmid of the indicated Rep variants. AAV2 Cap was co-expressed from the RepX plasmids as shown in panel (**A**). rAAV-GFP titers acquired with Rep2 were averaged and set to 1, n = 3. (**C**) Fold change in total capsids per ml cell culture lysate (vp/mL). HEK293T cells were transfected as described in panel (**B**). vp/mL titers acquired with Rep2 were averaged and set to 1, n = 2. (**D**) Fold change in packaging rate calculated from panels (**B**,**C**). Rates acquired with Rep2 were averaged and set to 1, n = 2. All data were consistently normalized to the Rep2 variant, as indicated by the red bar.

Conventional rAAV production systems relying on the Rep protein from AAV2 (hereafter called Rep2) have demonstrated efficacy in generating high viral titers across multiple AAV serotypes. However, the obtained viral titers remain notably lower than those of wild-type AAVs [33], and packaging efficiencies range from 5 to 50% of genome-containing capsids, depending on the production system used [33,34,35]. This prompted us to explore the potential of alternative naturally occurring Rep proteins. To this end, the complete *rep* open reading frames (ORFs) were sourced from published wild-type AAV sequences (1–13), detailed in the Materials and Methods section. Notably, a 16–25-base-pair DNA stretch connecting *rep* and *cap*, varying among AAV isolates, was included in the final constructs (Figure 1A). All *rep* ORFs were cloned upstream of the AAV2 *cap* gene and positioned behind a P5 mini-promoter element derived from wild-type AAV2. Rep protein expression from the various plasmid backbones was confirmed through Western blot analysis of transfected cell lysates (Figure S1A). In this manuscript, we refer to Rep proteins expressed from various serotypes by their respective serotype numbers for simplicity (e.g., the Rep protein from AAV5 will be referred to as Rep5).

To assess the functionality of the different Rep proteins in an AAV2-based vector system, a GFP reporter flanked by AAV2 ITRs was used. Adherent HEK293T cells were triple transfected with Rep_x_Cap (x = 1 to 13), rAAV-GFP, and an adenovirus helper plasmid. GFP expression served to determine transfection efficiency, which on average ranged between 57 and 66% (Figure S1B). Similarly, comparable cell viabilities between 70 and 78% were observed across all conditions, including the non-transfection control (Figure S1B). Cell lysates were utilized for various analytical measurements reflecting viral vector production: (i) viral genomes per ml (vg/mL), (ii) total AAV particles per ml (vp/mL), and (iii) the calculated ratio of (i) to (ii) representing the percentage of full capsids.

Significant differences were observed among the constructs in viral genomic titers, as determined by ddPCR analysis (Figure 1B). For instance, using Rep13, vg/mL titers improved to 112% compared to Rep2. Rep3 and Rep4 followed with 61% and 68%, respectively. Other Rep2 replacements resulted in a genomic titer reduction of >50%, notably Rep5 and Rep8, which reached 2–3% of the titers produced by Rep2.

To investigate whether lower genomic titers resulted from low capsid expression or a defect in genome packaging, the Enzyme-Linked Immunosorbent Assay (ELISA) was performed to measure total capsid formation. The data (Figure 1C) depict the fold change of detected capsids normalized to Rep2Cap2. AAV2 capsid yields in Rep3 and Rep13 constructs were at the same level as Rep2Cap2. Slightly reduced capsid amounts were seen for Rep4 and Rep12 constructs at 51% and 41%, respectively. Strikingly, Rep5 increased capsid production 10.4-fold relative to Rep2. All other constructs produced low levels of capsids at 14–26% of Rep2Cap2.

The ELISA and dPCR data were then used to calculate the packaging rate of all rAAVs with Rep1-13 compared to Rep2 (Figure 1D). Rep1, Rep9, Rep10, Rep12, and Rep13 were able to produce a genome packaging level similar to Rep2, ranging from 0.83- to 1.12-fold. The best genome packaging efficiencies were reached using Rep4, Rep7, and Rep11, showing 8–11% above standard conditions. In contrast, the lowest packaging rates were observed with Rep3 and Rep6. As expected, the Rep5 and Rep8 constructs with the very low genomic titers (Figure 1B) could package even fewer or no genomes at all.

### 2.2. DNA Family Shuffling (DFS) of AAV rep ORFs 1–13

**Figure 2:**
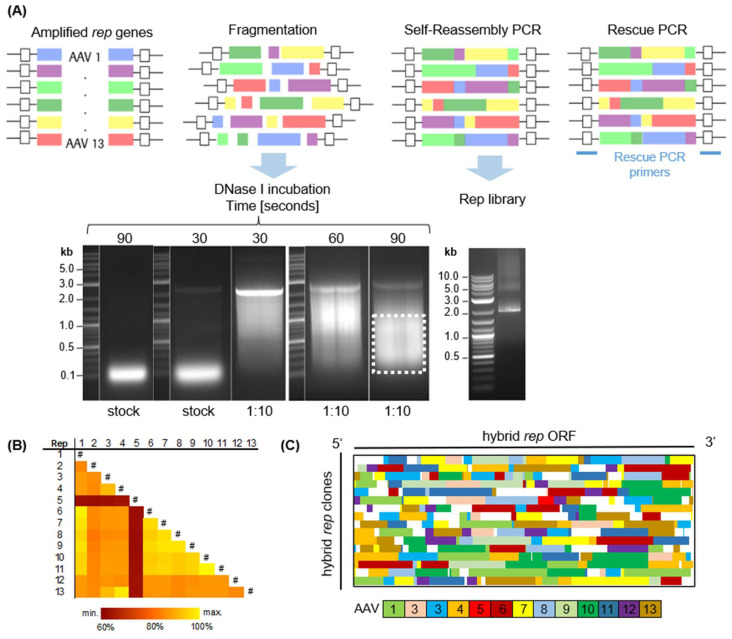
Generation of a chimeric *rep* library using DNA family shuffling. (**A**) Steps involved in DNA family shuffling. A pool of PCR-amplified *rep* ORFs undergoes a DNaseI digest under conditions indicated in respective agarose gel electrophoresis images. This involves varying times of incubation (30, 60, or 90 s) and DNaseI concentration (stock = 1 U/µL or 1:10 dilution). Fragments in the indicated range (100–1000 bp) are extracted from the gel and serve as input for a primerless, homology-based PCR. Finally, complete ORFs were amplified with a rescue PCR using primers binding outside of the shuffled region. (**B**) Percentage homology matrix of AAV *rep* ORFs 1–13. The # symbols in the figure mark the diagonal where each Rep variant is compared to itself, indicating 100% similarity (**C**) Hybrid rep library generated in this work, later denoted as library 0. Each line represents one clone from the 5′ to the 3′ end. Color blocks indicate the underlying parental *rep* sequences as shown by the color code at the bottom. “Maximum traces” were generated as described in Figure S3 and were used to build a graphical composition summary of each chimera. White annotated regions could not be exclusively assigned to one *rep* reference sequence.

To generate a highly diverse Rep library, DFS was employed as outlined in the Materials and Methods section. In brief, *rep* ORFs were PCR amplified and subsequently digested using DNaseI (Figure 2A). As previously described [36], the digestion conditions were optimized by experimenting with different incubation times and DNaseI concentrations. Subsequently, the digested fragments were extracted from the gel and reassembled in two consecutive PCRs to generate full-length *rep* hybrids, as demonstrated by the distinct 2kb-sized band in agarose gel electrophoresis (Figure 2A). The substantial homology exceeding 80% between the *rep* ORFs (except *rep5*) is crucial for the success of the first primerless PCR (Figure 2B). Indeed, the clonal composition of the library, as depicted in Figure 2C, showcases its high diversity, with representation from all parental serotypes and a various distribution of differently sized fragments. Rep5 is an exception, being underrepresented in the library and found in only one clone. Due to this high homology of the *rep* genes, some regions (white) were not clearly assignable to a single reference and therefore could not be annotated. The initial library displayed a total theoretical diversity of 3.3 × 10^6^ clones, estimated from the number of colony-forming units per mL.

### 2.3. Directed Evolution of Rep Proteins for Enhanced Functionality

**Figure 3:**
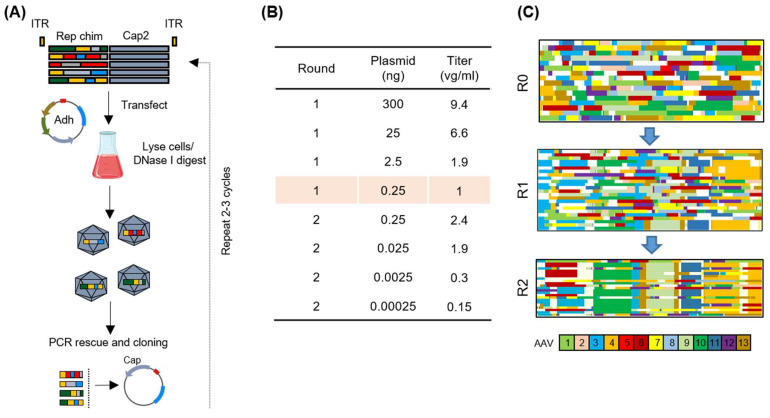
Directed evolution of Rep proteins for rAAV production. (**A**) Schematic representation of the directed evolution approach used. Suspension HEK293 cells were transfected with the chimeric *rep* library and an adenohelper plasmid for wild-type AAV2 production. Cells were lysed to recover AAV2 particles packaged with the chimeric *rep* ORFs. Primers binding outside of the shuffled region were used to PCR amplify the hybrid regions. Finally, chimeric *rep* ORFs were cloned into an acceptor plasmid with AAV2 *cap* for clonal assessment and initiation of second selection round. (**B**) Chimeric Rep/Cap plasmid amounts and corresponding genomic titers as fold changes. Titers obtained with the lowest DNA amount in round 1 (0.25 ng) were set to 1. (**C**) Clonal composition of *rep* hybrids after each selection round. Each line corresponds to one clone from the 5′ to 3′ end. Color blocks represent underlying *rep* reference sequences, as indicated with the color code at the bottom. R0 = primary, pre-selected library. R1/R2 = selection rounds 1 and 2.

Directed evolution stands out as one of the most potent methodologies for engineering proteins and entire organisms [37,38,39]. In our pursuit of applying this approach to Rep proteins, a host was selected to undergo cycling with the engineered Rep library, and two screening rounds were conducted (Figure 3A). In this process, the Rep plasmid library was transfected into suspension HEK293 cells alongside an adenovirus plasmid, facilitating wild-type AAV2 production. Unlike rAAV vector production, *rep* and *cap* were packaged into the AAV2 capsid. To establish a direct link between phenotype and genotype, we tested various DNA amounts (refer to Figure 3B), ranging from 0.00025 ng to 300 ng (equivalent to 0.00347 and 41,124 plasmids per cell, respectively). Subsequently, cells were harvested and lysed to release virus particles, followed by a DNaseI digest to ensure complete removal of residual plasmid DNA. Finally, packaged hybrid *rep* sequences were PCR amplified and cloned into the WT AAV2 acceptor plasmid for clonal assessment (Figure 3C) and initiation of the second selection round (Figure 3A). Interestingly, the genomic titer of the initial library was high enough to use the lowest plasmid amount tested (0.25 ng per 1 × 10^6^ cells). This is surprising in view of the randomness of DFS. After the second selection round, an increase in viral titers reflected an improved fitness of the library (compare 0.25 ng conditions in Figure 3B), most probably due to the enrichment of functional Rep hybrids.

Sequences from each selection round were aligned using MUSCLE, and clonal composition was assessed using an in-house macro. Strikingly, a shift in the clonal composition of the library was already observed after the first selection round and further accumulated after the second round. The most prominent clone exhibited an enrichment of *rep*3-, *rep*10-, *rep*13-, *rep*9-, *rep*11-, and *rep*4-derived sequences from the 5′ to the 3′ end (5/25). After the second round, another clone composed of the aforementioned *rep* parental sequences, except *rep*3, emerged, featuring a novel sequence derived from *rep*6 at the 5′ end, not observed in sequenced clones from round 1. To assess whether the enriched Rep clones are superior to Rep2, we selected variants from all selection rounds and first performed a small-scale, high-throughput functional assessment in 24-deep-well format.

### 2.4. Small- and Mid-Scale Assessment of Rep Hybrid Functionalities

**Figure 4:**
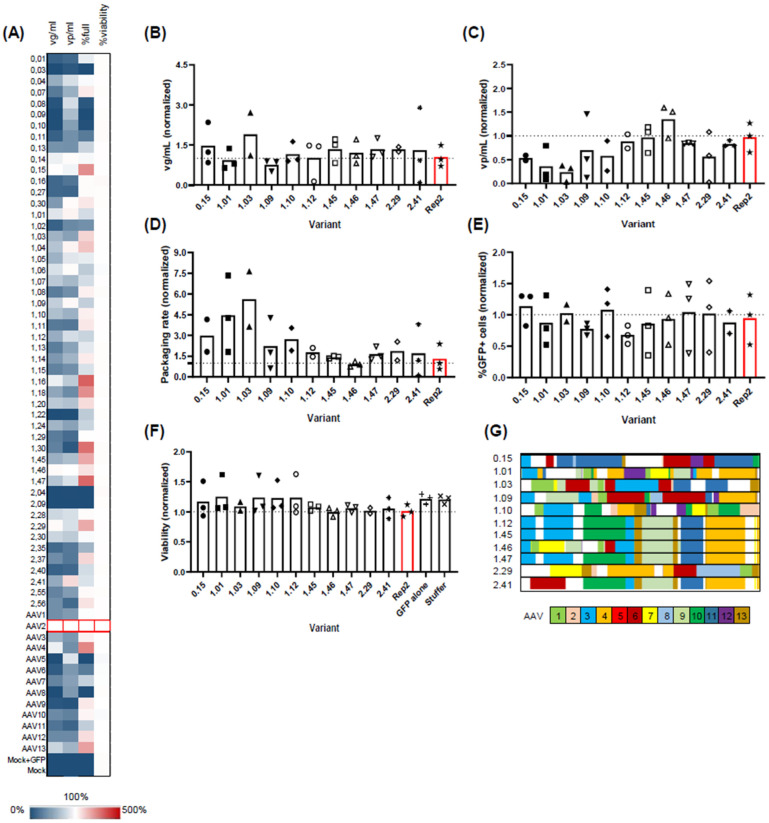
Functional validation of single *rep* hybrids. (**A**) High-throughput screening of Rep variants in a micro-scale setting (24 deep-well). Data from the indicated analytical measurements are represented as a heat map with Rep2-values set to 100% (white) and the highest limit corresponding to 500% of Rep2 levels (red); n = 2–3 biological replicates. Genomic titers ranged from 1 × 10^9^ to 2 × 10^10^ vg/mL, with values below 5 × 10^8^ vg/mL classified as negative. Capsid titers ranged from 1 × 10^10^ to 5 × 10^11^ vp/mL. (**B**–**F**) Testing of selected Rep candidates in mini-scale (30 mL shake flasks; n = 2–3 biological replicates). Indicated parameters were assessed: (i) genomic titer (panel (**A**); genomic titers ranged from 6 × 10^9^ to 2 × 10^11^ vg/mL); (ii) capsid titer (panel (**B**); capsid titers ranged from 2 × 10^11^ to 8 × 10^12^ vp/mL; (iii) packaging rate (ratio of (**A**) to (**B**)); (iv) functionality of resulting AAV vectors (% of GFP positive cells) after transduction with crude cell lysates (equal volumes were applied (1:10 dilution of stock)); and (v) cell viability at day of harvest. (**G**) Overview of the clonal composition of selected *rep* hybrids (5′ to 3′ end). Color blocks represent underlying *rep* reference sequences, as indicated with the color code at the bottom. The red bar in all images highlights the Rep2 wildtype variant.

Fifty variants were randomly selected from different selection rounds, with clone numbers starting at 0, 1, or 2 for the initial library or selection rounds 1/2, respectively (Figure 4A). Parental Rep variants 1–13 served as controls. Assessed parameters included viral genomic titers in the harvest (vg/mL), capsid titer (vp/mL), packaging rate, and cell viability.

In line with observations in adherent HEK293 cells, Rep3, 4, and 13 outperformed other natural Rep variants, displaying vg/mL titers close to Rep2 (55.8%, 91.6%, and 75.8% of Rep2 titers, respectively). Furthermore, Rep4 and 13 demonstrated an enhanced packaging rate with 3.0- and 2.5-fold increases (median values), aligning with our findings.

As anticipated, the majority of clones from the initial library exhibited minimal to no viral genomic titers. Exceptions included variants 0.04, 0.14, and 0.15 (3/14 clones), which exhibited vg/mL titers above 50% of Rep2 titers. In contrast to the genomic titer, the vp/mL titer appeared less affected, with 10/14 variants from the initial library displaying either good (above 50%) or medium (above 30%) vp/mL titers compared to Rep2.

Contrary to the pre-selected library, clones from the first and second selection rounds were predominantly functional, yielding vg/mL titers ranging from 11.42% to 108% and vp/mL titers ranging from 6.3% to 120% of Rep2 levels. The packaging rate, the vg/mL-to-vp/mL ratio, was higher than Rep2 for several clones: 0.15, 1.03, 1.04, 1.16, 1.18, 1.20, 1.30, 1.45, 1.47, 2.29, 2.37, and 2.56. However, clones 1.30, 2.37, and 2.56, despite showing a favorable packaging rate, exhibited low viral vg/mL titers (below 50% of Rep2) and were consequently excluded from further consideration. Notably, cell viability remained unaffected, ranging from 96% to 108% of Rep2.

From the small-scale screen, we selected 11 variants for further validation at a mid-scale level (30 mL shake flask; Figure 4B–F). Most tested variants produced similar genomic titers to Rep2 (Figure 4B), but some showed significant differences in vp/mL titers (Figure 4C), i.e., the total viral capsids (e.g., variants 0.15, 1.01, and 1.03). This resulted in a shift towards a favorable packaging rate, which is the ratio between the two parameters (Figure 4D). We also assessed the functionality of the AAV2 capsids produced using these different Rep variants by packaging a GFP-encoding genome into the constructs and transducing adherent HEK293A cells with the resulting crude lysates at multiple dilutions. As shown in Figure 4E, only subtle differences were observed that reflect the variations in the genomic titers, confirming the ability of all Rep hybrids to produce functional rAAV vectors. Likewise, no significant differences were observed in producer cell viability (Figure 4F). We then performed Sanger sequencing analysis of the clones and assessed the clonal composition using an in-house macro (Figure 4G). We found three identical sequences (1.12, 1.45, and 1.47) representing the leading clone after the first selection round. Importantly, all three plasmid preps behaved nearly identically in our mid-scale validation, serving as internal benchmarks for the assay. Another two clones, 1.46 and 2.41, were very similar to the leading clone, differing only in their 5′ sequence. All other clones showed a distinct, chimeric nature.

### 2.5. Functional Assessment of Leading Clones 2.41 and 1.03 in the AMBR15 and 250 Fermentation Systems

**Figure 5:**
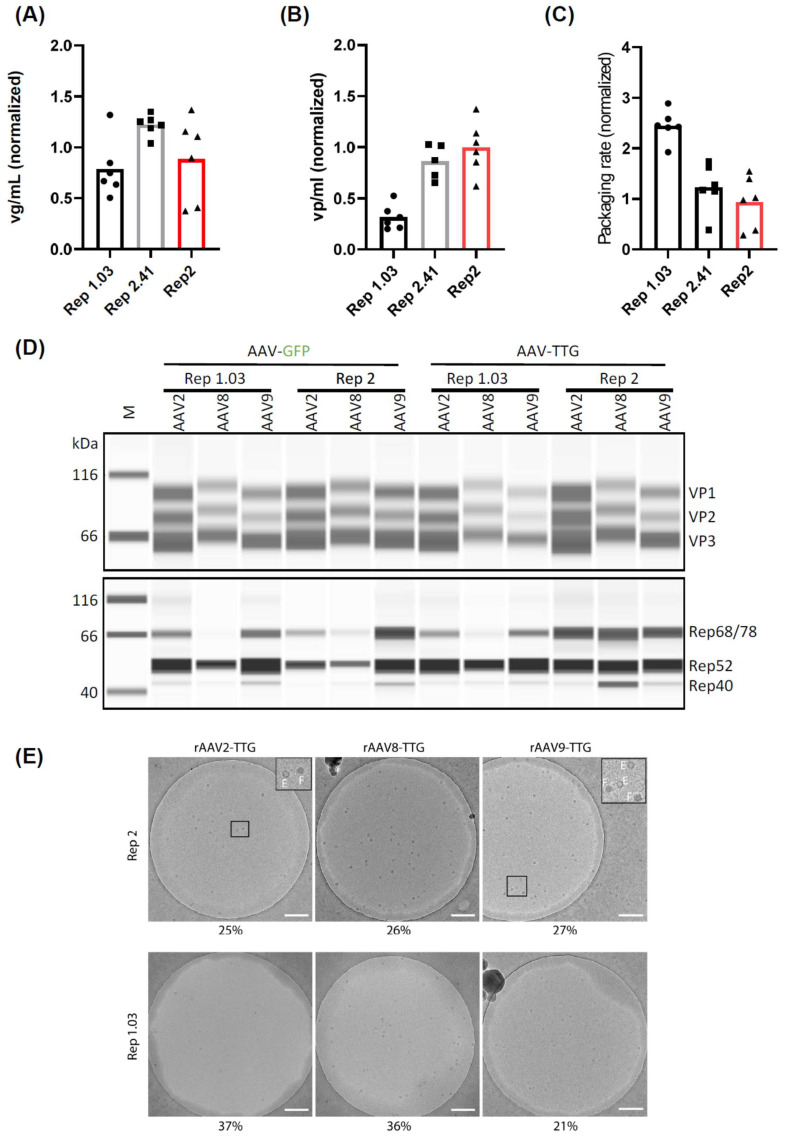
(**A**–**C**): Production of rAAV-GFP with the indicated Rep variants or Rep2 control in the AMBR15 system. Assessed parameters: (i) genomic titer (panel (**A**)), (ii) capsid titer (panel (**B**)), (iii) packaging rate (ratio of (**A**) to (**B**)). n = 3 biological replicates. The red bar in all images highlights the Rep2 wildtype variant. (**D**) Western blot analysis using the JESS Simple Western™ system. Cell lysates from the indicated rAAV productions in the AMBR250 system were analyzed for capsid protein expression (VP1-VP3; top) or Replication protein (Rep) expression (bottom). A semi-quantitative analysis is provided in Tables S2 and S3 TTG = therapeutic transgene. (**E**) Cryo-EM analysis of the indicated rAAV productions in AMBR250 purified using small-scale AAVX columns. For each sample, a representative image is shown. Two zoom-in areas show examples of typical empty (E) and full (F) particles. Scale bars = 200 nm.

Mini- and mid-scale high-throughput screening approaches are attractive, as they require only a small amount of material. However, translating data from such systems into large-scale fermenters can be challenging due to significant differences in culturing conditions. Therefore, we sought to validate our findings in mini and midi bioreactor systems—the AMBR15 and AMBR250. We first conducted a side-by-side comparison of Rep1.03, Rep2.41, and Rep2 in the AMBR15 system, using GFP as a transgene (Figure 5A–C). Consistent with our previous findings, Rep2.41 slightly increased the viral genomic titers (Figure 5A) but did not affect the packaging rate (Figure 5C). Conversely, Rep1.03 did not alter the viral genomic titers but increased the packaging rate by decreasing the total capsid amount by more than 50% (Figure 5B).

Next, we combined our leading Rep variant, 1.03, with the capsid of two other AAV serotypes (8 and 9). These constructs were tested for rAAV production in the AMBR250 system using two different transgenes—GFP and a therapeutic gene (TTG). Consistent with our earlier results in the mini- and mid-scale, Rep1.03 increased the amount of full AAV2 capsids by 2-fold and 3.5-fold for GFP and TTG, respectively (Table S1). The packaging rate for AAV8 was also increased, with a 1.67- and 2.3-fold increase for GFP and TTG, respectively. However, this did not apply to AAV9, which showed a general decrease in vp/mL but no increase in the packaging rate (Table S1).

To further understand the observed decrease in capsid formation, we performed Western blot analysis of VP and Rep protein expression (Figure 5D). For AAV2, we did not observe a decrease in the major capsid protein (VP3) expression using Rep1.03 compared to Rep2 (Figure 5B and Table S2) with both transgenes. Therefore, the sharp decrease in capsid formation cannot be explained by a low availability of capsid protein. However, the slight transgene-dependent decrease in AAV8 and AAV9 VP3 expression might explain the noted decrease in capsid formation. Rep protein expression (Figure 5D and Table S3) varied between the conditions, with a trend toward decreased large Rep68/78 expression in conditions containing Rep1.03.

Finally, we aimed to confirm our findings using an orthogonal method that does not rely on ratio calculations. Therefore, we purified a small fraction of our AMBR250 lysates using an AAVX affinity resin that binds both full and empty particles, thereby preserving their original ratio in the cell lysates. The viral genome titer (vg/mL) of these preparations was quantified using three distinct primer/probe sets targeting the 5′ end, 3′ end, and middle of the genome (Figure S4). Notably, we observed highly similar titers when comparing the simplex and multiplex assays, highlighting the robustness of our dPCR protocol. Additionally, a multiple-occupancy analysis was performed to estimate the percentage of intact genomes. The analysis revealed that differences between Rep2 and Rep1.3 were either absent or small and depended on the transgene (Figure S4). Primer/probe sequences targeting the commonly used CMV-GFP-BGH transgene are provided in Table S5. Next, EM analysis of the purified lysates was performed, and the percentage of full particles was estimated using manual image analysis (Figure 5E). As expected from our previous data, the percentage of full capsids did not improve for AAV9 in the case of the therapeutic transgene. However, Rep1.03 increased the percentage of full capsids to 37% and 36% for AAV2 and AAV8, respectively (compared to 25% and 26%, respectively, using Rep2). Similarly, it increased the percentages from 21 to 25% for AAV2 and from 15 to 18% for AAV8 in combination with a GFP transgene instead of the therapeutic transgene (Table S4).

## 3. Discussion

The widespread availability of gene therapies is hindered by the persistently high costs of producing rAAVs [1,2,40]. Although ongoing technical innovations aim to cut costs, certain aspects of the production process itself continue to pose challenges. Essentially, three key components are needed: (i) the cargo, containing a transgene cassette flanked by ITRs; (ii) AAV Rep and Cap proteins supplied in trans; and (iii) genes from a helper virus, usually adenovirus. Strategies to enhance this system include plasmid engineering (e.g., minicircles/nanoplasmids [41,42], doggybone^TM^ [43,44]), process optimization [45,46], and creating stable cell lines [47,48] or adenovirus [49]/herpesvirus- [50] and baculovirus- [51,52] based systems that overcome plasmid dependency. Importantly, all of these methods maintain the integrity of the protein components with modification to the expression cassettes themselves to fit into the respective systems.

A major focus in both academic and industrial laboratories has been the engineering of Cap proteins [53]. The main goal is to guide the vector toward specific tissues/cells, enhancing specificity and ultimately reducing production costs. Some engineered capsids naturally show higher vector titers than their parental counterparts, especially those originating from directed evolution approaches [54]. Two other shifted ORFs within *cap*, namely *aap* [55] and *maap* [56], have been linked to vector stability and production. Due to their relatively recent discovery, efforts to engineer these ORFs are still less explored [57,58] compared to the extensively studied *rep* ORF, which plays a crucial role in genome replication and packaging during viral vector production [10,14].

Currently, the Rep2 variant from the AAV2 serotype is commonly used in rAAV production systems, partly due to historical reasons and the reported superiority of this Rep [26,28]. Our results in both adherent and suspension cells support these previous observations, extending them to Rep isolates not studied before, as demonstrated by our side-by-side comparison of 13 naturally occurring Rep proteins. Overall, Rep3, Rep4, and Rep13 outperformed other Rep variants in the genomic titer of AAV2-ITR-flanked genomes in both cell types. A notable point in this context is that we found a close phylogenetic relationship between the complete *rep* 3, 4, and 13 ORFs on DNA (Figure S2A,B) and protein level (Figure S3B–D), supporting the functional similarities reported in our work. On the other hand, Rep5 and Rep8 yielded the lowest genomic titers reported in this study. This is in line with previous work that has demonstrated that Rep5 does not recognize AAV2 ITRs, rendering it unable to package AAV2-based genomes [59]. Notably, Mietzsch and colleagues [30] recently suggested correcting the AAV8 *rep* nucleotide sequence in the VR-A and VR-B region, which led to enhanced AAV8 VP expression. It would be interesting now to test the impact of this correction in our constructs.

Also, in concordance with previously reported data for Rep1 [26,30], 3 [26], 5 [26], 6 [30]. and 8 [30], capsid expression is low when using Rep proteins other than Rep2 (in our study below 50% of Rep2 AAV2 capsid yield; see Figure 1C and Figure 4A). Despite exceptions like Rep5 outperforming Rep2 in VP expression in adherent cells, the overall trend is in favor of Rep2. Importantly, in the two aforementioned studies, the Rep proteins were combined with their respective Cap sequences, whereas in this study the *cap* gene was kept constant (AAV2-based). This makes a direct comparison between the studies difficult. Notably, however, is the strategy used by Grimm and colleagues to enhance capsid expression in all backbones, which relies on the use of the MMTV promoter instead of p5 to drive Rep expression [60]. This modification might increase Cap2 expression in our backbones as well. In another study by Rabinowitz and colleagues [25], the *rep2* gene was kept constant and combined with AAV capsids of different serotypes. This also resulted in low capsid expression, which was then reversed by appending stretches from the respective *rep* sequences at the 5′-end of the *cap* genes, highlighting again the promise of Rep hybrids in optimizing rAAV production.

In the above-mentioned study by Mietzsch and colleagues [30], low Cap expression of AAV6 and AAV8 capsid proteins when combined with their respective Rep proteins was linked to the DNA sequence of the region encoding the zinc finger domain. Swapping that region with the same region of *rep*2 rescued VP protein expression. This is interesting when compared to the study by Rabinowitz et al. [25], who replaced the same region in *rep*2 with sequences derived from other *rep* variants, *rep*3, *rep*4, and *rep*5, to produce the respective AAV serotypes, increasing particle production up to 1000-fold. However, the yields using Rep2 were lower than in other reports that persistently showed good compatibility of Rep2 with different AAV capsids. Importantly, in this latter study, a small serotype-specific stretch between *rep* and *cap* was included. In a following study, the importance of this junction sequence in constructing a hybrid rep5/Cap8 helper was shown [61]. Based on these findings, we likewise added the respective sequences to our different constructs. Again, these differences in expression constructs make comparisons between studies difficult, and for now, it remains an open question how the whole 3′-DNA region contributes to the observed effects. Another recent study showed the impact of the 5′-DNA region, involving the OBD in increasing DNA packaging ability. Here, inverting four amino acid residues at the N-terminus of Rep6 to their counterpart in Rep2 restored Rep6 expression and ability to package AAV2 ITR-based genomes to packaging rates similar to Rep2 (but lower genomic titers) [20].

Given the different properties of the Rep proteins, roughly classified into (i) good viral genomic titers (Rep2, Rep3, Rep4, Rep13), (ii) good capsid titers (Rep2, Rep5), and (iii) good packaging rates (Rep4, Rep13, Rep12), we speculated that a directed evolution approach for these proteins might result in new variants with a combination of desired properties. To this end, we applied DFS to diversify the *rep* ORF, a method previously used to create novel enzymes [39], fluorescent proteins [62], and viral capsids [37,38]. A prerequisite for this method is a high sequence similarity of the genes used [36]. Indeed, the *rep* ORFs with more than 80% sequence similarity (Figure 2B and Figure S2B) were suitable substrates for random DFS, as exemplified with the diverse clonal composition (Figure 2C) and theoretical library diversity (>10^6^ clones). As expected, *rep*5, which shares less than 60% similarity with the other *rep* ORFs, was underrepresented. Methods to increase the incorporation of less homologous sequences have been described and would allow the creation of even more complex libraries [63].

We then subjected the library to a selection process by performing cycles of wild-type AAV2 production in suspension cells. Surprisingly, after just one selection round, there was an increase in specific domains’ presence and an enrichment of clones, which became even more pronounced after the second selection round (see Figure 3C). It is noteworthy that none of the clones contained sequences from the superior *rep2*, and the domain composition remained highly chimeric, with the most dominant clone composed of sequences derived from seven parental *rep* ORFs. Also noteworthy were the high titers of the unselected library, reflecting a high plasticity of the Rep proteins. The observed enhancement in viral titers after each selection round indicates an increase in the fitness of the underlying clones, as described earlier for AAV capsid libraries [64]. Functional validation of single clones confirmed the accumulation of viable *rep* ORFs, as the majority of the unselected clones were dysfunctional with some exceptions (see Figure 4A). A number of clones were transferred for functional validation and further evaluation from micro- to mini-scale culture formats. Here, good translatability between the scales was observed. All selected clones performed comparably well or better than Rep2 in packaging a GFP reporter flanked by AAV2 ITRs. However, major differences were observed in genome packaging efficiency, where several clones (0.15, 1.01, 1.03, 1.10) outperformed Rep2 while maintaining high viral genomic titers. This aligns with observations by Mietzsch et al. [30], who reported Rep hybrids that mainly increased the packaging efficiency of AAV6, AAV8, AAV9, and AAvrh.10. Importantly, in this latter study, the identified Rep hybrids did not increase AAV2 packaging efficiency. We also confirmed that none of the Rep hybrids affected the transduction ability of the resulting viral vectors. This is expected, as Rep is not incorporated into the viral capsid.

Finally, we moved two candidates (1.03 and the dominant clone 2.41) into the AMBR15 [65] and eventually AMBR250 [66] fermentation systems, which are micro and mini bioreactors, respectively, that allow automated control of culturing conditions and offer great scalability to larger bioreactor settings. Here, we confirmed the superior packaging rates for clone 1.03 (2.5-fold on average). Moreover, we tested Rep 1.03 with two further AAV serotypes (8 and 9) and two different transgenes (GFP, therapeutic transgene). We consistently report higher packaging rates for AAV2 and AAV8, but not AAV9, reinforcing our previous observations in the micro and small scale and confirming the high promise of Rep 1.03 for rAAV production. Interestingly, this Rep variant outperformed the leading clone 2.46 and displayed a very chimeric and distinct domain composition compared to the other chimeras (underlying serotypes 1, 2, 3, 6, 7, 9, 11 and 13; Figure S3A). Also of note is its close phylogenetic relationship in the helicase domain to Rep3, Rep4, and Rep13, the most promising naturally occurring variants (Figure S3C). The other two protein domains—OBD and ZF—cluster with other Rep variants, e.g., Rep7, but without a clear trend (Figure S3 panels B and D). This contrasts with Rep2.41, which contains more sequences from *rep*9, 10, and 4 (Figure 4G). It remains an open question why Rep1.03 did not show clonal selection despite its superior packaging efficiency. The answer might be found in the kinetics of vector amplification or the superiority of particle production with Rep 2.41, which results in more viral capsids available for packaging viral genomes—a process known to occur post-assembly [67]. As Rep 2.41 has no benefit over Rep2, there is a need for refinement of the selection strategy to detect more Rep proteins with desired features.

In summary, we have created a highly complex Rep library from which we have selected a new chimeric Rep variant demonstrating superior packaging abilities for both AAV2 and AAV8. This variant can now be further optimized through directed engineering approaches, such as promoter selection and transcriptional control. Finally, it is noteworthy that viral ITRs play a detrimental role in the wt virus replication cycle and in recombinant virus production [68]. Hence, another layer of optimization can be added by combining our new hybrid Rep with differently sized transgenes flanked by either naturally occurring ITR sequences [69] or synthetic derivatives thereof [70].

## 4. Material and Methods

### 4.1. Cloning Procedures

#### 4.1.1. Cloning of Replication-Competent and -Incompetent Acceptor Plasmids for Rep ORF Cloning

The pWTAAV2_2xBsmI_Cap2 acceptor plasmid contains two AAV2 ITRs flanking a *ccdb* gene and the AAV2 *cap* ORF. The *ccdb* gene is of bacterial origin and part of a type II toxin–antitoxin system and has been used here to increase cloning efficiency. This gene is flanked by inverted BsmI sites to allow the seamless cloning of *rep* ORFs. A gene block containing the whole sequence was ordered from GeneWiz, NJ, USA. The replication-incompetent pAAV2_2xBsmICap2 (lacking AAV2 ITRs) was generated from pWTAAV2_2xBsmI_Cap2 by PCR amplifying the complete region between AAV2 ITRs and cloning (SnabI/EcoRV) into an acceptor plasmid [71] that contains a mini p5 promoter proximal to *rep* and a distal full-length p5 at the end of the *cap* ORF (distal to *rep*).

#### 4.1.2. Cloning of Rep1–13 ORFs and Hybrid Rep Library

Gene blocks of the *rep* genes originating from AAV serotypes 1–13 were ordered at GeneArt (Thermo Fisher Scientific, Waltham, MA, USA). The *rep* gene sequences were derived from the NCBI GenBank entries: *rep1*: NC002077; *rep2*: NC001401; *rep3*: U48704; *rep4*: NC001829; *rep5*: NC006152; *rep6*: AF028704; *rep7*: NC006260; *rep8*: NC006261; *rep9*: AX753250; *rep10*: AY631966; *rep11*: AY631965; *rep12*: DQ813647; *rep13*: EU285562. To allow the cloning of Rep3 using BsmI, we introduced a silent mutation A>G at nucleotide position 1827. Rep gene blocks were flanked with sequences from the AAV2 genome to enable the use of common primers for amplification (Primer_fwd: AGC GCA TTG CGT AGA ATA C, Primer_rev: CTG CGT GGA CAC TCA CTT). The resulting PCR amplicons were separated using agarose gel electrophoresis and purified with the QIAquick PCR Purification Kit (Qiagen, Hilden, Germany) according to manufacturers’ instructions. These PCR amplicons served as input for both individual *rep* ORF amplification and DNA family shuffling. Two other sets of primers were used to introduce restriction enzyme recognition sites into the *rep* amplicons (for both individual *rep*1–13 and hybrid *rep* ORFs): Primer_fw_AscI_BsmI: TGA AGC GGC GCG CCG AAT GCG GGA GGT TTG AAC GCG C; Primer_rev_PacI_BsmI: TTA GTA TTA ATT AAG AAT GCG AGC CAA TCT GGA AGA TAA CC). AcsI/PacI double digest was performed to clone PCR amplicons into an empty plasmid backbone containing only an ampicillin resistance gene and a multiple cloning site. From this plasmid subcloning of the individual *rep* ORFs or amplified *rep* library was performed using BsmI into the final pAAV2_2xBsmICap2 or pWTAAV2_2xBsmI_Cap2 plasmid, respectively.

### 4.2. DNA Family Shuffling of rep ORFs

DNA family shuffling was performed as previously described [36]. Briefly, PCR-amplified *rep* gene blocks (see cloning of *rep* 1–13 ORFs and hybrid *rep* library) were pooled (total 4 µg) and subjected to a DNaseI digest using the conditions shown in Figure 2A. Fragments between 100 and 1000 bp were extracted from the agarose gel and purified with the NucleoSpin Gel and PCR Clean-up kit according to manufacturer’s instructions (Macherey-Nagel, Düren, Germany). From the digest, 500 ng were used as template in a primerless PCR (98 °C for 30 s; 40 cycles of 98 °C for 10 s, 42 °C for 30 s, and 72 °C for 45 s; and final elongation at 72 °C for 10 min), followed by an amplification of the final ~1900 bp hybrid *rep* ORFs using the above-mentioned primers Primer_fw_AscI_BsmI/Primer_rev_PacI_BsmI that bind to common regions outside of the hybrid *rep* ORFs (98 °C for 30 s; 40 cycles of 98 °C for 10 s, 64 °C for 30 s, and 72 °C for 60 s; and final elongation at 72 °C for 5 min). The final amplicons were cloned as described above.

### 4.3. Cell Culture

Expi293F^TM^ suspension cells were grown in Expi293™ Expression Medium (Thermo Fisher Scientific) and maintained at 37 °C with 8% CO_2_ incubation following supplier’s instructions. HEK293T and HEK293A cells were grown in Dulbecco’s modified Eagle’s medium (DMEM) with high glucose (Thermo Fisher Scientific) supplemented with fetal bovine serum (Merck, Darmstadt, Germany), sodium pyruvate, and GlutaMAX (both Thermo Fisher Scientific). Cells were grown at 37 °C with 5% CO_2_ incubation.

### 4.4. AAV Vector Production

Small-scale production of rAAVs in adherent HEK293T cells was performed as previously described using a standard triple-transfection protocol [72], including a pRep_x_Cap plasmid (x = 1–13 or hybrid Rep variant), the adenohelper (Takara Bio, Kusatsu, Japan), and a transgene flanked by AAV2 ITRs (either GFP or a therapeutic gene).

For rAAV production in suspension cells, Expi293F^TM^ cells were grown at a density of 1.8 × 10^6^ cells per ml in the following formats and volumes: Microscale 4 mL cultures, 24-well plate; Miniscale 30 mL shake flasks; AMBR15, 15 mL bioreactor; AMBR250, 250 mL bioreactor. Transfection was performed using the same plasmids listed above for rAAV production in adherent cells and according to a previously described protocol [73]. For experiments in adherent cells, all assays were performed with crude lysates generated by centrifuging cells (at 800× *g*, 10 min) and resuspending the pellet in PBS. The cell suspension was transferred to thin-walled PCR tubes and placed at 4 °C in a bath sonicator Q700MPXC Microplate Horn System (QSonica, Newtown, CT, USA). Samples were sonicated for 3 min with 30 s on/off increments at 25% amplitude (intensity). In contrast, assays from suspension cultures were performed from crude lysates generated by chemical lysis (CG110, 1%). For transduction assays, however, crude lysates were also generated by sonication as the lysis conditions interfered with the assay.

### 4.5. AAV Vector Purification

Crude cell lysates were centrifuged for 5 min at 1000× *g* at room temperature and subsequently purified on columns containing 80 µL of AAVX resin per column (Biotage, Uppsala, Sweden; PTR-91-80-33). Purification was performed according to the manufacturer’s instructions using buffers recommended in the manufacturer’s protocol. To increase particle concentration for Cryo-EM analysis, final elution fractions and individual columns were re-used for 3 runs of purification. PBS was used as a negative control during the purification process.

### 4.6. Transduction Assays

For transduction of HEK293A cell monolayers with rAAV, cells were seeded in 96-well plates one day prior to transduction at a density of 2.0 × 10^4^ cells per well. Next, 10 μL of rAAV crude lysates (stock or diluted 1:10 in PBS) were added to each well. After 72 h, the medium was removed and the cells subjected to flow cytometry analysis.

### 4.7. Droplet Digital (dd) and Digital (d) Polymerase Chain Reaction

To determine the amount of encapsidated viral genomes per mL (vg/mL), droplet digital (dd) PCR was performed. Ten µL of crude lysates (generated by sonication or chemical lysis) were subjected to a pre-treatment with DNaseI RQ1 RNase-free #M610A (Promega, Madison, WI, USA) to remove plasmid DNA, followed by Proteinase K digest (New England Biolabs, Ipswich, MA, USA) to open the rAAV capsids as previously described [74]. Droplets were generated in the QX200 AutoDG Droplet Digital PCR System according to manufacturer’s instructions (Bio-Rad Laboratories, Hercules, CA, USA). PCR was performed in the C1000 Thermal Cycler (Bio-Rad Laboratories) and final quantification of viral genomes in the Droplet Reader (Bio-Rad Laboratories). Data presented in Figure S4 were generated using the QIAcuity digital (dPCR) system, also following the manufacturer’s instructions (Qiagen). The same pre-treatment steps as described above (DNase I digestion, Proteinase K digestion) were applied to remove contaminating plasmid and genomic DNA. Control singleplex dPCR reactions were conducted to evaluate the performance of individual primer/probe sets. Subsequently, all three primer/probe sets—targeting the 5′ end, 3′ end, and mid-region of the genome—were combined in a triplex reaction.

The QIAcuity Software Suite (version 2.5.0.1) provides a downloadable multiple-occupancy CSV file. This file incorporates mathematical approximations based on Poisson distribution statistics to estimate the percentage of intact viral genomes. For both viral titers and genome integrity analyses, the average value from four different dilutions was calculated for each replicate. Final reported values represent the mean of two independent replicates. Primer sets used to quantify the rAAV-GFP genome used in this work can be found in Table S5. As the therapeutic transgene is a non-public sequence; primers to target this area are not shared.

### 4.8. Flow Cytometry Analysis of Transduction and Transfection

Efficiencies of transfection and transduction of cells were evaluated via flow cytometry (FACSCanto II, BD Biosciences, Franklin Lakes, NJ, USA) using the green fluorescent protein (GFP). Therefore, transduced HEK293A cells were detached using 25 μL of 0.25% Trypsin/EDTA and then resuspended in 175 μL PBS with 1% bovine serum albumin (BSA). Next, 100 μL of the cell suspension was measured by flow cytometry. Expi293^TM^ suspension cells used for recombinant or wild-type AAV production were also analyzed for their transfection efficiency by including a control transfection with GFP. Flow cytometry measurements were performed at the harvest day, i.e., 72 h after transfection. Therefore, 100 μL of the cell suspension was directly pipetted into a 96-well plate and used for the flow cytometry measurement. 

### 4.9. Automated Western Blot Analysis

Capillary electrophoresis (Jess) was used for separation of proteins. Therefore, suspension cells were centrifuged for 3 min at 300× *g*, washed with PBS, and lysed using 100 µL RIPA buffer, supplemented with 1× Pierce™ Protease Inhibitor Mini tablets, EDTA-Free (Thermo Fisher Scientific). Lysates were incubated on ice for 20 min, sonicated for 3 min, and then centrifuged at 20,000× *g* for 15 min. Supernatants were collected, and protein concentrations were determined using the BCA protein assay kit (Thermo Fisher Scientific). Prior to electrophoresis, protein samples were prepared according to manufacturer’s instructions (ProteinSimple, part of Bio-Techne, San Jose, CA, USA). Final concentration was set to 0.5 mg/mL.

The following primary antibodies were used for detection of Rep proteins (anti-AAV2 Replicase mouse monoclonal, 303.9, lyophilized, purified; PROGEN) and Cap proteins (anti-AAV VP1/VP2/VP3 rabbit polyclonal (VP51), serum; PROGEN), both at a 1:100 dilution in Antibody Diluent 2 (ProteinSimple, part of Bio-Techne). For the detection of the housekeeping protein Glycerinaldehyd-3-phosphat-Dehydrogenase (GAPDH), a monoclonal mouse antibody (GAPDH (D4C6R) Mouse mAb #97166; Cell Signaling, Danvers, MA, USA) was used at a 1:25 dilution in Antibody Diluent 2 (ProteinSimple, part of Bio-Techne).

The following Separation Modules were used for the detection of Rep proteins (12–230 kDa Separation Module) and Cap proteins (66–440 kDa Separation Module).

The following Detection Modules were used for the detection of Rep proteins (Anti-Mouse Detection Module; ProteinSimple, part of Bio-Techne) and Cap proteins (Anti-Rabbit Detection Module; ProteinSimple, part of Bio-Techne). The total protein detection kit (ProteinSimple, part of Bio-Techne) was used for normalization of protein concentrations (in Figure 5D) and prepared according to manufacturers’ instructions.

The RePlex kit (ProteinSimple, part of Bio-Techne) was used to allow binding of the total protein detection antibody and was prepared according to manufacturers’ instructions (ProteinSimple, part of Bio-Techne).

For the separation of Rep and Cap proteins in Figure 5D, the Jess system was programmed according to the following protocol: Separation Time, 28 min: Separation Voltage, 375 volts; RePlex Purge Time, 30 min; Biotin Labeling Time, 30 min; Antibody Diluent Time; 5 min; Primary Antibody Time, 60 min; Secondary Antibody Time, 30 min; Total Protein HRP Time, 30 min.

For the separation of Rep and GAPDH in Figure S1A, the Jess system was programmed according to the following protocol: Separation Time, 30 min: Separation Voltage, 375 volts; Biotin Labeling Time, 30 min; Antibody Diluent Time; 5 min; Primary Antibody Time, 60 min; Secondary Antibody Time, 30 min.

Chemiluminescent signals were detected and quantified using the Compass software (version 6.3.0) (ProteinSimple, part of Bio-Techne). Protein bands were identified based on the molecular weight ladder and peak intensities were normalized using the total protein detection kit (ProteinSimple, part of Bio-Techne).

### 4.10. Enzyme-Linked Immunosorbent Assay (ELISA) and Electrochemiluminescence Immunoassay (ECLIA)

ELISA was performed to detect total AAV capsids in producer cell lysates. Here, the CaptureSelect™ anti-AAV affinity reagents were used according to the manufacturer’s instructions. In brief, Pierce streptavidin-coated 96-well plates (Thermo Fisher Scientific) were incubated with 100 μL/well Biotin Anti-AAVX Conjugate #7103522100 (1:4000 in 1× PBST, Thermo Fisher Scientific) for 2 h at RT. rAAV lysates and a standard control vector AAV2-CMV-GFP (Virovek, CA, Hayward, USA) were diluted using PBST (1×). Samples were measured in duplicate and at different dilutions to fit in the limited linear range of the standard curve. For detection, 100 μL HRP Anti-AAVX Conjugate #7303522100 (1:10,000 in 1× PBST, Thermo Fisher Scientific) was applied to each well and incubated for 1 h at RT. Development reagent Ultra TMB ELISA substrate (Thermo Fisher Scientific) was used at a volume of 100 μL/well (incubation 15 min). The reaction was stopped with 100 μL of 1M HCl and the OD measured at a wavelength of 450 nm using the microtiter plate reader Infinite 200 PRO (Tecan, Maennedorf, Switzerland).

The ECLIA assay was used for increased sensitivity in the small-scale experiments (with expected lower titers) and for testing of Rep1.3 for the production of different AAV isolates. Therefore, a heterogeneous sandwich assay based on AAVX (for AAV2 and AAV8 production) or anti-AAV9 antibody for AAV9 production (both Thermo Fisher Scientific) was used.

### 4.11. Electron Microscopy

For negative staining EM, electron microscopy grids (T600H-Cu 698 l/inch Hex. mesh Thin Bar; EMS) were coated with an in-house ~2 nm carbon film by floating the carbon on H_2_O and letting the water level drop till the carbon covered the grids. After at least 2 days of drying, the grids were used. Three µL of sample was incubated on a glow-discharged carbon coated grid for 30 s, followed by 2 steps of washing with H_2_O, one step of washing with UAc 2%, and 30 s incubation in UAc 2% staining solution. After the final blotting step, the sample was left to dry.

For cryo-electron microscopy, 3 µL of sample was incubated on a glow-discharged carbon-coated Quantifoil (Quantifoil—R1.2/1.3, 300, Cu + 2 nm, Jena, Germany) or carbon-coated Lacey (UC-A on Lacey, 400 Mesh Cu, TED PELLA, INC., Redding, CA, USA) grid for 60 s. It was subsequently blotted for 2.5 or 3 s, followed by plunging the grid into liquid ethanol at −180 °C using a Leica EM GP automated plunging device (Leica Microsystems, Vienna, Austria).

Grids were loaded into a Jeol JEM-1400 Plus transmission electron microscope operating a Lab6 electron source at 120 kV. Electron micrographs were recorded on TVIPS XF416 4000 by 4000 pixel charge-coupled device camera (Tietz Video and Image Processing System, Gauting, Germany). The negative staining images were used to check the presence of whole particles, ascertain that the particle concentration is suitable for cryo-EM, and check for aggregations and impurities prior to freezing the samples. Cryo-EM datasets were recorded at a nominal magnification of 30,000× yielding pictures with a pixel size corresponding to 0.3914 nm at the specimen level. Cryo-EM grids were imaged using low-dose mode.

Depending on the particle concentration, 25–80 images per sample were imported into the EMAN2 software package (version 2.91) [75] where all the viral particles that were observed on the thin carbon were picked and sorted manually to determine the ratio between empty and full capsids. Some intermediately filled particles were observed and counted as empty since they lacked the complete genome, avoiding the difficulty of manually sorting into intermediate categories.

## Data Availability

Data are contained within the article.

## Supplementary Materials

The following supporting information can be downloaded at: https://www.mdpi.com/article/10.3390/ijms26031319/s1.
