# Supplementary material for: Improved Recombinant Adeno-Associated Viral Vector Production via Molecular Evolution of the Viral Rep Protein
Source: Int J Mol Sci. 2025 Feb 4;26(3):1319. doi: 10.3390/ijms26031319 (PMC11818820; doi:10.3390/ijms26031319)
Supplement: Supplementary file 1 [file ijms-26-01319-s001.zip › ijms-3262448-supplementary.pdf]

(A)

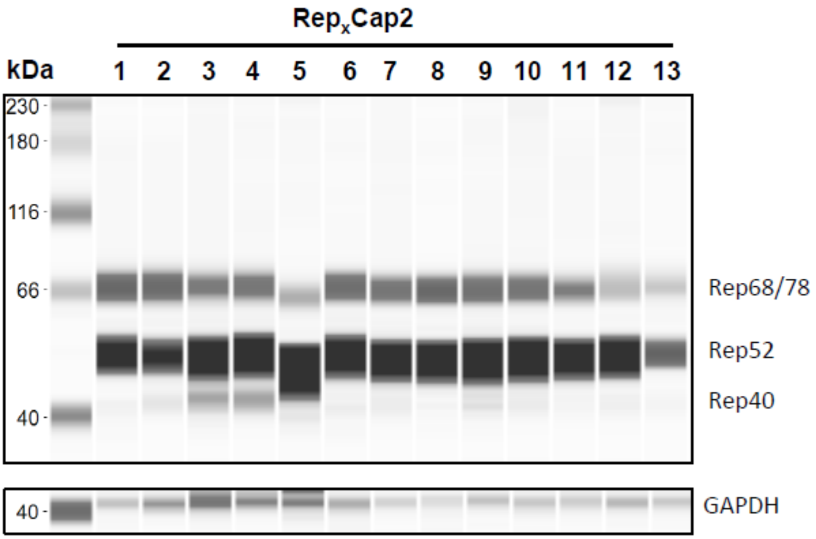

(B)

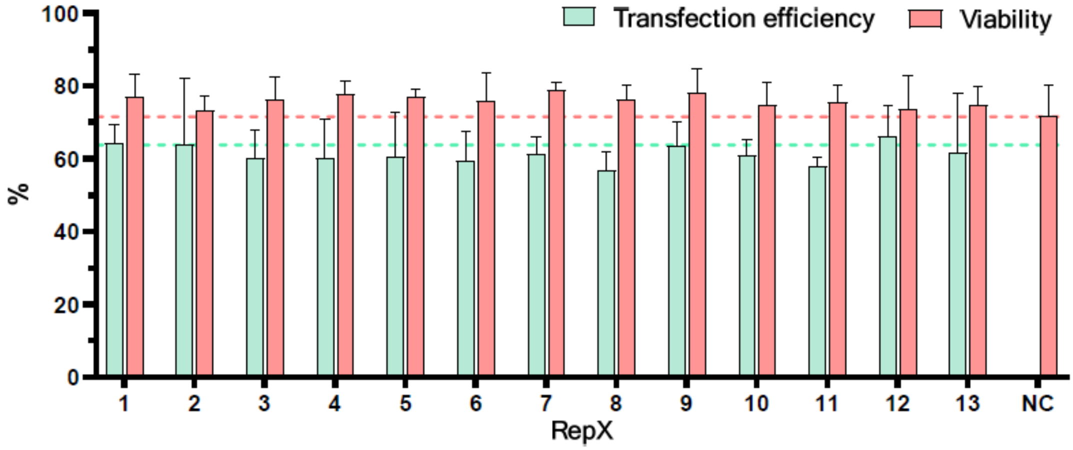

Assessment of AAV Rep variants in HEK293 cells. A) Western Blot analysis using the JESS Simple Western™ system. Expi293F™ suspension cells were transfected with the indicated Rep_XCap2 plasmids (x = 1–13), alongside the adenohelper and an AAV-GFP reporter plasmid. Cells were harvested three days post-transfection, and lysates were analyzed for Rep protein expression (top panel) and GAPDH expression (bottom panel) as a loading control. The expected Rep protein variants are indicated. **B)** Cell viability and transfection efficiency were assessed for the samples used in the analyses shown in Figure 1B–D. HEK293T cells were harvested in PBS three days post-transfection, followed by viability and flow cytometry analyses. NC = non-transfection control.

Figure S1

(A)

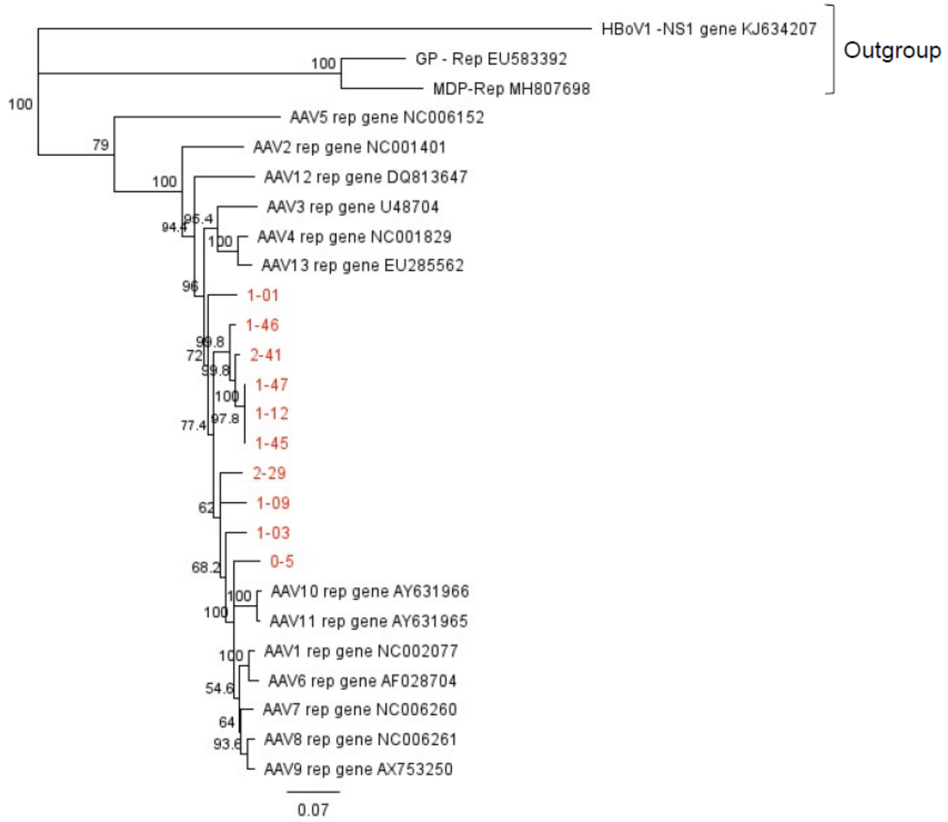

(B)

	AAV1	AAV2	AAV3	AAV4	AAV5	AAV6	AAV7	AAV8	AAV9	AAV10	AAV11	AAV12	AAV13	C0-15	C1-01	C1-03	C1-09	C1-10	C1-12	C1-45	C1-46	C1-47	C2-29	C2-41	GP - Rep	HBoV1 - NS1	MDP - Rep
AAV1																											
AAV2	85																										
AAV3	88	86																									
AAV4	88	85	91																								
AAV5	61	61	61	62																							
AAV6	95	82	84	84	58																						
AAV7	94	82	84	84	58	96																					
AAV8	96	84	86	87	61	92	94																				
AAV9	96	84	87	87	61	93	94	98																			
AAV10	95	85	88	87	61	91	92	95	95																		
AAV11	95	85	88	87	61	91	92	95	95	99																	
AAV12	87	85	87	86	61	83	84	86	86	87	87																
AAV13	87	86	91	97	62	83	84	86	87	87	87	85															
C0-15	87	78	82	82	55	89	90	87	88	90	90	80	82														
C1-01	84	80	83	86	56	86	86	83	83	83	84	82	86	90													
C1-03	87	80	84	84	56	90	90	86	87	86	87	80	83	93	91												
C1-09	93	86	89	90	61	90	90	92	92	92	92	86	90	85	87	85											
C1-10	89	89	90	90	62	87	87	89	89	90	90	89	90	83	83	85	88										
C1-12	90	85	91	93	62	87	88	90	91	91	91	87	93	85	86	85	92	93									
C1-45	90	85	91	93	62	87	88	90	91	91	91	87	93	85	86	85	92	93	100								
C1-46	92	86	89	92	62	89	90	92	93	92	92	86	92	85	87	88	93	91	97	97							
C1-47	90	85	91	93	62	87	88	90	91	91	91	87	93	85	86	85	92	93	100	100	97						
C2-29	92	87	90	92	62	89	89	92	92	91	91	86	91	85	86	88	92	91	91	91	92	91					
C2-41	92	86	90	93	62	89	89	91	92	92	92	87	93	85	86	86	92	92	98	98	98	98	92				
GP - Rep	48	49	48	48	46	49	49	48	48	48	48	48	48	47	48	48	48	48	48	48	48	48	48	47.743			
HBoV1 - NS1	32	33	33	33	31	32	32	32	32	32	32	32	33	30	31	30	33	33	33	33	33	33	33	32.826	27.851		
MDP - Rep	47	48	47	47	45	48	47	46	47	47	47	47	47	47	47	47	47	48	47	47	46	47	47	46.712	83.386	27.895	

Phylogenetic and distance analysis of Rep hybrid clones. A) Phylogenetic analysis of the indicated *rep* variants (colored red). The evolutionary history was inferred in Geneious Prime by using the Neighbor-Joining algorithm based on the Tamura- Nei model. The percentage of replicate trees in which the associated taxa clustered together in the bootstrap test (500 replicates) are shown next to the branches. The scale bar represents the average number of nucleotide substitutions per site.
B) Nucleotide identity matrix. The percentage of nucleotide identity among the indicated nucleotide sequences.

Figure S2

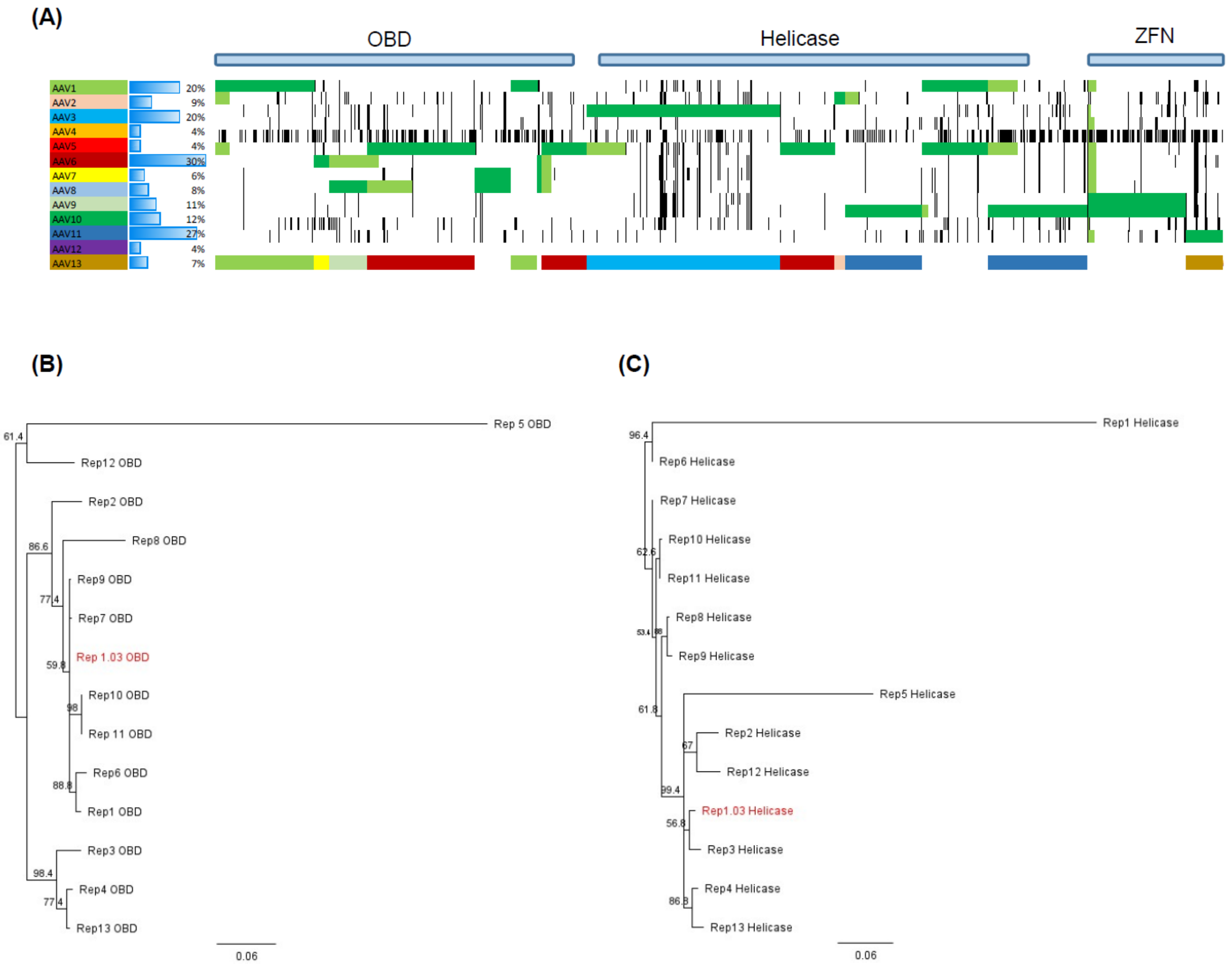

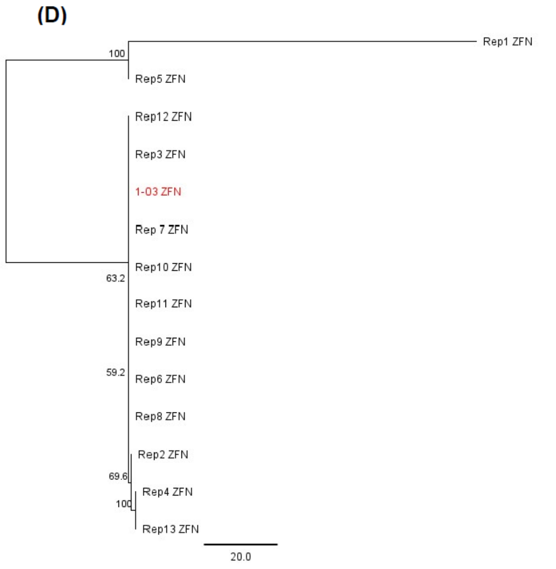

Composition and phylogenetic analysis of Rep 1.03. A) Chimera 1.03 from selection round 1 was aligned to the 13 parental *rep* references using MUSCLE (DNA alignment). Mismatches to the references at single nucleic acid positions are colored in black. Matching regions are shown in green and were ranked according to their length. A "maximum trace" – colored in dark green – reflects the highest probability of origin of a particlular region. If several traces were identified, the longer stretch was colored in dark green and the others in light green. Blue bars reflect the percentage of each reference sequence in the clone composition. Above the alignment the approximate location of the underyling Rep domains is shown.

B-D) Phylogenetic analysis of the indicated Rep domains. OBD = origin binding domain (AA residues 1-225); Helicase (AA residues 225-490); ZF = Zinc finger domain (AA residues 531-625). Hybrid Rep variant 1.03 is colored red. The evolutionary history was inferred in Geneious Prime by using the Neighbor-Joining algorithm based on the Jukes-Cantor model. The percentage of replicate trees in which the associated taxa clustered together in the bootstrap test (500 replicates) are shown next to the branches. The scale bar represents the average number of amino acid substitutions per site.

Figure S3

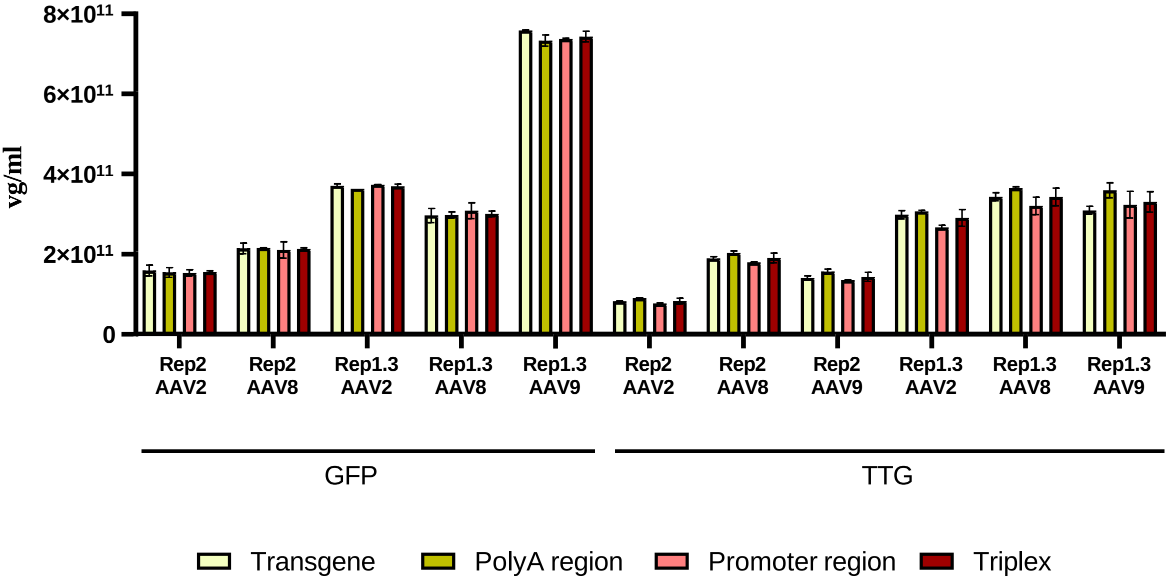

(B)

Transgene	Rep Hybrid	Capsid	Intact genomes (Ratio)
**GFP**	Rep2	AAV2	1
**GFP**	Rep1.3	AAV2	1.004
**GFP**	Rep2	AAV8	1
**GFP**	Rep1.3	AAV8	1.005
**TTG**	Rep2	AAV2	1
**TTG**	Rep1.3	AAV2	0.82
**TTG**	Rep2	AAV8	1
**TTG**	Rep1.3	AAV8	0.91
**TTG**	Rep2	AAV9	1
**TTG**	Rep1.3	AAV9	0.80

Genomic titers and genome intactness using Rep2 and Rep1.3. A) Absolute quantification of rAAV titers using dPCR. A total of 15 mL of the indicated rAAVs, produced in the AMBR 250 system, were purified using AAVX affinity resin. Singleplex and multiplex titer analyses were conducted using primer/probe sets targeting different regions of the recombinant genome: the 3′ end, 5′ end, and the middle. **B)** Estimation of viral genome intactness. The genome intactness of the rAAV preparations shown in panel A was assessed using the multiple-occupancy analysis feature of the QIAcuity Software Suite (version 2.5.0.1), as described in the Materials and Methods section. Percentages derived using Rep2 were averaged and normalized to a reference value of 1.

Figure S4

Table S1. Production of rAAV encoding for GFP or a therapeutic transgene (TTG) in an AMBR250 system

Parameter	vg/ml		vp/ml		%full	
Transgene	**GFP**	**TTG**	**GFP**	**TTG**	**GFP**	**TTG**
Rep1.03_Cap2	0.7	0.5	0.53	0.2	2.0	3.5
Rep1.03_Cap8	1.1	1.25	0.61	0.5	1.67	2.3
Rep1.03_Cap9	ND	0.65	ND	0.4	ND	1.3

[a]Genomic (vg/ml) and capsid (vp/ml) titers were assesed using ddPCR and ECLIA, respectively. The calculated ratio of both parameters represents the percentage of full capsids. Data were normalized to the Rep2-control (set to 1).

Table S2. Semi-quantitative Western blot analysis of Cap protein expression (shown in Figure 5)

Protein	VP1		VP2		VP3	
**Transgene**	**GFP**	**TTG**	**GFP**	**TTG**	**GFP**	**TTG**
Rep1.03-AAV2	0.99	0.86	0.89	0.67	1.07	0.99
Rep1.03-AAV8	0.77	0.67	0.75	0.65	0.81	0.65
Rep1.03-AAV9	0.80	0.57	0.70	0.50	0.77	0.56

[b]The area under the peak was calculated using the Compass for SW Version 6.3.0 and corrected to the total protein content. Data of each condition were normalized to the respective Rep2 control construct.

Table S3. Semi-quantitative Western blot analysis of Rep protein expression (shown in Figure 5)

Condition	Rep 1.03-AAV2		Rep 1.03-AAV8		Rep 1.03-AAV9	
**Transgene**	**GFP**	**TTG**	**GFP**	**TTG**	**GFP**	**TTG**
Rep40	2.9	0.78	1.5	0.48	0.9	0.74
Rep52	4.8	1.25	2.03	0.6	1.5	1.3
Rep68/78	1.58	0.35	0.32	0.12	0.44	0.39

[c]The area under the peak was calculated using the Compass for SW Version 6.3.0 and corrected to the total protein content. Data of each condition were normalized to the respective Rep2 control construct.

Table S4. Overview of the % full of the rAAV harvest as depending on Rep, Cap and transgene combination

Rep	Cap	Transgene	Nr. of analyzed AAVs	% Full
1.3	2	GFP	637	25
2	2	GFP	2376	21
1.3	2	Therapeutic	413	37
2	2	Therapeutic	672	25
1.3	8	GFP	1411	18
2	8	GFP	1220	15
1.3	8	Therapeutic	1972	36
2	8	Therapeutic	1437	26
1.3	9	GFP	ND	ND
2	9	GFP	2724	22
1.3	9	Therapeutic	1171	21
2	9	Therapeutic	917	27

Table S5. Primer/probe sets used for singleplex and multiplex dPCR analysis of rAAV titer

**Target**	**Primer/Probe name**	**Sequence (5′→3′)**
5’ end	5’GFP_CMVenh5_F	TTGACGTCAATGGGTGGAGT
	5’GFP_CMVenh5_R	CGGGCCATTTACCGTCATTG
	5’GFP_CMV3nh5_P	ACATCAAGTGTATCATATGCCAAGTACGC
3’ end	3’GFP_BGHpolA_F	[CY5]CATTGTCTGAGTAGGTGTCATTC[BHQ2]
	3’GFP_BGHpolA_R	TGCCTGCTATTGTCTTCCCA
	3’GFP_BGHpolA_P	[HEX]CCTCCCCCTTGCTGTCCTGC[BHQ1]
Middle	eGFP_mid_F	TAGCCGCTACCCTGATCATA
	eGFP_mid_R	TAAAGAAGATGGTCCGCTCC
	eGFP_mid_P	[FAM]TCTTTAAGTCCGCTATGCCAGAAGG[BHQ1]
